# The Effectiveness of Common Interventions in the Management of Sickle Cell Disease in Primary Care Settings: A Review

**DOI:** 10.7759/cureus.44780

**Published:** 2023-09-06

**Authors:** Rheiner N Mbaezue, Adetoro T Okafor, Bernard I Nkwocha, Chidalu N Ibeneme, Amoge C Opara, Darlington E Akahara, Okelue E Okobi

**Affiliations:** 1 Health Services, Department of Health, Cape Town, ZAF; 2 Public Health, University of Limpopo, Polokwane, ZAF; 3 Epidemiology and Public Health, University of Minnesota School of Public Health, Minneapolis, USA; 4 Internal Medicine, University of Utah College of Medicine, Salt Lake City, USA; 5 Public Health, University of Toledo, Toledo, USA; 6 Medicine and Surgery, Biologic Delivery Technologies, Reno, USA; 7 Medicine, Windsor University School of Medicine, Cayon, KNA; 8 Family Medicine, Larkin Community Hospital Palm Springs Campus, Hialeah, USA; 9 Family Medicine, Medficient Health Systems, Laurel, USA; 10 Family Medicine, Lakeside Medical Center, Belle Glade, USA

**Keywords:** strategies, interventions, primary care, sickle cell trait, anemia, sickle cell disease

## Abstract

Sickle cell disease (SCD), a chronic condition that affects men and women equally, continues to present a public health burden in the United States due to its associated morbidity and complications. Despite advances in medical knowledge and the design of novel therapies for managing the disease, its burden remains compounded because of increasing rates of immigration arising from global displacements and economic unrest in many countries. We thus conducted a comprehensive literature review of publications from 2000 to 2022 to gather guidelines on managing SCD, with a search through four databases, PubMed, Embase, Google Scholar, and Cochrane; 42 articles met the final inclusion criteria after the full-text article screening process. In the United States healthcare system, primary care physicians (PCPs) are generally providers who cater to the lifelong management of chronic medical conditions, SCD not being an exception. While more SCD patients now present to primary care clinics, many PCPs still lack the confidence and adequate experience necessary to manage the condition effectively. The gap created by the shortage of PCPs extensively equipped to provide comprehensive SCD care leads to poor health outcomes for patients. It is imperative now more than ever to continue to raise awareness about this condition at the provider level, to ensure that patients receive well-rounded care to improve their quality of life and clinical outcomes. Providing up-to-date knowledge about existing and novel therapies and/or modalities of SCD treatment would undoubtedly equip the PCPs with self-assurance to manage the condition adeptly. Thus, we explore various public health interventions such as hydroxyurea therapy, pneumococcal vaccination, penicillin therapy, iron chelation therapy, and clinical decision support tools that have been implemented in primary healthcare settings and shown to be effective in improving SCD care. We also discuss recent advancements that can lead to improved outcomes for SCD patients in the future.

## Introduction and background

Sickle cell disease (SCD) is a group of inherited red blood cell (RBC) disorders in which RBCs become crescent- or sickle-shaped, leading to acute and chronic clinical complications [1]. It is the most common inherited disorder of hemoglobin and arises from inheriting at least one "sickle" hemoglobin gene, known as hemoglobin S (HbS), a variant of the hemoglobin, beta (HBB) gene. SCD is predominant among people living in North and South America, the Caribbean, Central America, India, Saudi Arabia, Sub-Saharan Africa, and Mediterranean countries like Turkey, Greece, and Italy. Approximately 100,000 people with SCD live in the United States, including an estimated 15,000 patients in the Midwest [1,2].

Hemoglobin comprises four protein chains, two alpha chains, and two beta chains, each with a ring-like heme group containing an iron atom (in the ferrous state, Fe2+). Oxygen binds reversibly to these iron atoms and is transported through blood. The normal type of hemoglobin in adults described above is hemoglobin A (HbA). Different globin genes encode each type of globin subunit. Genes for the alpha chain are on chromosome 16, and genes for the beta chain are on chromosome 11. Sickle cell hemoglobin (HbS) arises from the substitution of adenine by thymine at the sixth position of the beta-globin gene, replacing glutamic acid with valine in the same place of the beta-globin chain.

HbS causes RBCs to take the shape of a sickle in stressful/deoxygenated conditions instead of their standard biconcave shape. This abnormal shape causes several manifestations from two primary mechanisms: (i) Blockage of small blood vessels (postcapillary venules), with subsequent Ischemia and infarction, and (ii) Activation of the immune system (reticuloendothelial cells), leading to their premature destruction [3,4].

**Figure 1:**
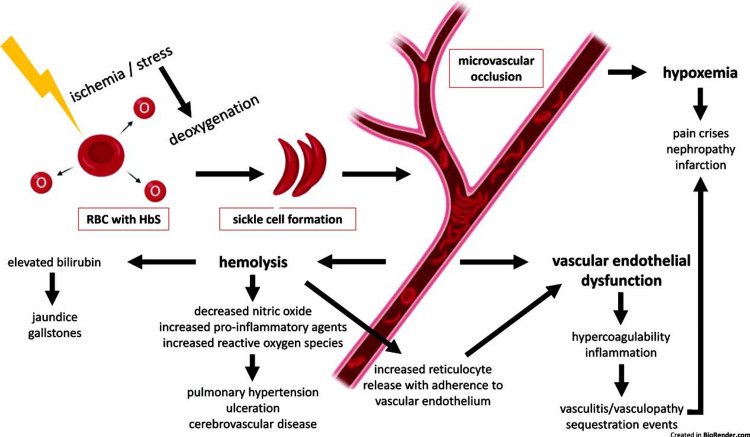
Pathophysiology of SCD manifestations Image Source: Solomon et al., 2022 [5]; Used with permission. SCD: sickle cell disease; HbS: sickle cell hemoglobin

**Figure 2:**
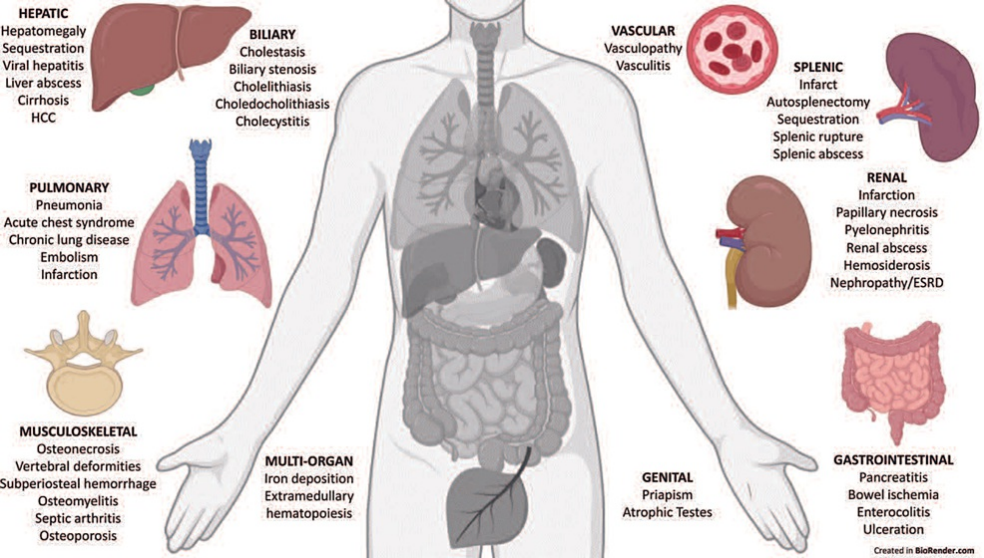
Manifestations of SCD Image Source: Solomon et al., 2022 [5]; Used with permission. SCD: sickle cell disease; HCC: hepatocellular carcinoma

The manifestations of SCD are bone pain, anemia, jaundice, stroke, recurrent infections (due to functional asplenia/autosplenectomy), priapism, failure to thrive, loss of concentrating ability of the kidneys, poor vision (from retinal vascular changes), pulmonary hypertension, venous thromboembolism, chronic leg ulcers, gallstones, etc. (Figures 1-2).

SCD cell disease can occur in different forms. The major forms are described in Table 1. The minor forms (hemoglobin sickle D disease (HbSD), hemoglobin SE (HbSE) disease, and hemoglobin SO (HbSO)) are rare, and their severity can vary [4,6].

**Table 1:** Major forms of SCD HbS: sickle hemoglobin; HbC: hemoglobin C; HbA: hemoglobin A; SCD: sickle cell disease

Sickle Cell Anemia	Hemoglobin SC	Sickle Cell Trait	Sickle Cell Beta Thalassemia
Homozygous inheritance. Most common in Africa and individuals of African origin.	Co-inheritance of HbS and HbC. HbC arises by the substitution of glutamic acid by lysine at the sixth position of the beta-globin chain. It is a milder form of SCD.	HbS and HbA co-existence. Sickling does not occur commonly but can occur in extreme conditions, e.g., high altitude severe dehydration etc.	Beta-thalassemia and HbS co-existence. Symptoms can be severe. Thalassemia is a condition that occurs when the body does not make enough of the globin chains of hemoglobin.

SCD is among the most common disorders identified via universal newborn screening in the United States. It was once a childhood disease as many patients died before adulthood; however, today, over 95% of children diagnosed at birth survive to young adulthood but still suffer early mortality by age 40 [2]. The shift in mortality from childhood to adulthood is accompanied by an increase in severe morbidity from chronic organ damage, including cerebrovascular, cardiopulmonary, and renal disease among teens and young adults [7].

Complex chronic diseases such as SCD can be challenging, especially for PCPs with limited experience, ancillary support, and time to manage SCD-related pain and complications [2]. Few PCPs are well-informed about the guidelines and confident in delivering evidence-based care to the SCD population [2].

We conducted a comprehensive literature review spanning 22 years, from 2000 to 2022, to gather guidelines on managing SCD. Four databases, PubMed, Embase, Google Scholar, and Cochrane, were scanned using the keywords "Sickle cell disease," "primary care," and "interventions," which were combined using Boolean operators (AND, OR). Forty-two articles met the final inclusion criteria after the full-text article screening process. 

## Review

Management and treatment of SCD

In 2014, the National Heart, Lung, and Blood Institute (NHLBI) of the National Institutes of Health published evidence-based guidelines for managing pediatric and adult SCD, including recommendations for increasing hydroxyurea use in all age groups [8]. Hydroxyurea is a daily oral medication that can change the clinical course of SCD [9] and improve survival for pediatric and adult SCD patients [10]. It causes significant reduction in pain and vaso-occlusive crises, frequency of hospitalizations, need for blood transfusions, and other complications [11]. Its ability to increase fetal hemoglobin levels and modify the disease course makes it a valuable therapeutic option. Despite the known clinical benefits, only 20-30% of eligible adults in the United States are prescribed this treatment [12]. Provider knowledge and comfort level with hydroxyurea are barriers that have limited patients' access to the therapy [10]. Many providers have also cited concerns about the long-term side effects of this therapy for SCD patients [10].

Pneumococcal vaccination has proven effective in preventing life-threatening infections (e.g., pneumonia and meningitis) in SCD patients [13]. Patients with SCD have impaired immune systems and are prone to infection even with vaccination, as they may not maintain adequate immunogenicity. Evidence has shown, however, that pneumococcal vaccines reduce the risk of invasive pneumococcal disease (IPD) [14]. For this reason, routine and vaccine-specific schedules for patients with SCD must be encouraged by primary physicians.

Penicillin prescription, as a prophylactic measure, has been widely recommended to reduce the risk of bacterial infections, particularly in children with SCD [15].

Iron overload resulting from chronic blood transfusions necessitates iron chelation therapy to remove excess iron from the body [16]. This approach is vital to preventing organ damage caused by iron deposition.

The utilization of CDS tools in primary care settings has gained significance in recent years. These tools assist healthcare providers in making evidence-based decisions by providing clinical guidelines, drug interactions, and dosing recommendations tailored to individual patients [17].

Effectiveness of hydroxyurea in the management of SCD

Hydroxyurea is a safe medication, even among patients as young as six to nine months of age. Although mild adverse effects such as transient gastrointestinal symptoms (abdominal pain, vomiting, and diarrhea), as well as hyperpigmentation of skin, palms, and nails, have been reported [18], there is insufficient evidence regarding the long-term risks [19]. For many years, it has been the only drug available to modify the severity of SCD. However, there is insufficient evidence to support its long-term benefits in preventing the chronic complications of SCD, as well as a paucity of data on its efficacy in the management of patients with other forms of SCD, such as those with HbSC genotype [20].

Regardless of the above, it has effectively reduced vaso-occlusive crises, acute chest syndrome, blood transfusions, and frequency and duration of hospitalizations in SCD patients. In their study, Ofakunrin et al. found a 46.3% reduction in painful crises among participants aged 4-17 years [21]. By causing a decrease in WBC and platelet counts, hydroxyurea consequently reduces the risk of vaso-occlusive events among patients while causing increased energy levels and improved appetite following therapy. Ballas also reported that hydroxyurea prevents life-threatening neurological events in children and adults at risk of primary stroke [19].

Effectiveness of the pneumococcal vaccine in the management of SCD

Pneumococcal disease, caused by *Streptococcus pneumoniae *or pneumococcus, is a significant cause of morbidity and mortality in SCD patients. *S. pneumonia* is an encapsulated gram-positive bacterium that causes infections like pneumonia, meningitis, otitis media, sinusitis, bacteremia, and sepsis. Transmission is by respiratory droplet or auto-inoculation and is often in the background of a viral respiratory infection. Infection with encapsulated organisms like *S. pneumonia* can be severe and life-threatening in patients with SCD. This is because functional asplenia from infarctions caused by their hemoglobinopathy results in a suboptimal immune system.

Pneumococcal disease causes approximately 150,000 hospitalizations annually in both children and adults in the United States, and the incidence rises in late fall, winter, and early spring. *S. pneumonia *is a leading cause of IPD and non-invasive diseases in patients with SCD, including meningitis, sepsis, pneumonia, and acute otitis media [22,23]. Routine and specific pneumococcal vaccinations are recommended to reduce the risk of severe infection in SCD patients [24]. Two vaccine types are currently used in the United States: (i) Pneumococcal conjugate vaccine (PCV-13/Prevnar13®) introduced in 2010. It consists of a pneumococcal bacteria polysaccharide linked to a diphtheria protein, enhancing adaptive immune response (T cell-dependent immune response) [25], and (ii) Pneumococcal polysaccharide vaccine 23 (PPSV-23/Pneumovax®), which consists of polysaccharide capsules from 23 serotypes most virulent in developed countries. It was licensed for use in 1983, covering 11 serotypes more than PCV-13. It elicits an IgM-mediated immune response with no generation of memory cells, thus requiring a booster dose due to the waning immunity after about five years of the last dose [25].

Numerous studies have shown a significant decrease in IPD after introducing pneumococcal vaccinations [26,27]. PPSV-23 is ineffective in children < 2 years [28], and as such, the Advisory Committee on Immunization Practices (ACIP) of the CDC recommends routine PCV-13 be given in four dose series to children with SCD < 2 years and an additional two doses of PPSV-23 given at five-year intervals. Although the FDA has approved the higher valent pneumococcal conjugate vaccines, PCV-15 and PCV-20 for adults ≥ 18 years old to prevent IPD [29], these have not been approved for patients with SCD. For adults with SCD ≥ 19 years (including ≥ 65 years), sequential interval vaccination with PCV-13 followed by PPSV-23 is recommended [24]. Yee et al. demonstrated in a 10-year cohort analysis that there was a significant decline in bloodstream infections due to pneumococcal vaccination in addition to lowering IPD [23]. Other studies also showed that almost 70% of patients with SCD maintained immunogenicity to IPD for up to five years following vaccination [30,31].

However, although there is overwhelming evidence for the role of vaccination in reducing the incidence of IPD in patients with SCD, the currently available vaccines do not cover up to 60% of serotypes that cause infection in SCD patients [30]. This is because pneumococcal serotypes constantly change in virulence, dependent on age, region, socioeconomic status, etc. [28]. For this reason, investigators are exploring serotype-independent options to have a broader pneumococcal serotype coverage.

Effectiveness of penicillin in the management of SCD

Before the introduction of pneumococcal vaccines, penicillin prophylaxis was a standard approach to reducing the risk of pneumococcal infections in individuals with SCD. Brown et al. conducted one study that evaluated the use of pneumococcal vaccine and penicillin prophylaxis in SCD [32]. This study assessed the practices of healthcare providers in three African countries. Although the study focused on the pneumococcal vaccine, it provided valuable insights into the use of penicillin prophylaxis. Rankine-Mullings and Owusu-Ofori assessed the efficacy of various antibiotic regimens, including penicillin, in reducing the risk of pneumococcal infections [33]. They concluded that penicillin significantly reduces the risk of pneumococcal infections and related complications in patients with SCD. In another study, Cober and Phelps discussed the rationale behind penicillin prophylaxis, the recommended dosage, and the impact on infection rates [15]. Their findings reemphasized the effectiveness of penicillin in reducing the incidence of pneumococcal infections and its potential to improve the overall health outcomes of children with SCD. Overall, penicillin prophylaxis plays a crucial role in treating SCD, particularly in reducing the risk of pneumococcal infections, as several studies suggest that penicillin effectively decreases the incidence of infections and related complications in children and individuals of different age groups.

Effectiveness of iron chelation therapy in the management of SCD

Blood transfusion is associated with many adverse effects, especially alloimmunization, and excess iron stores, which are usually very severe in patients with SCD [4]. Iron overload from repeated blood transfusions can lead to organ failure and death, hence the need for chelation therapy. Chelation therapy aims to balance the rate of iron accumulation from blood transfusion by increasing iron excretion in urine or feces with chelators [34]. Chelation therapy is started in patients with SCD based on the number of transfusions, the degree of iron deposition in the liver and the heart, and the amount of hepatic and cardiac dysfunction present. Therapy is usually started after one or two years of transfusion in chronically transfused patients when the serum ferritin exceeds 1000-1500 mcg (ng/ml) or the liver iron is > 3-5 mg/g dry weight [4].

Baseline testing before chelation includes audiology, ophthalmology, and pregnancy tests in females. There are three iron-chelating agents: deferiprone, deferoxamine, and deferasirox. Deferasirox is an orally available iron chelator approved in 2005 for individuals two years of age and older with iron overload. It decreases liver iron concentration with tolerable side effects in patients with SCD. Major adverse effects include gastrointestinal symptoms, a reversible dose-dependent rise in serum creatinine, and occasional liver dysfunction. Deferiprone is an orally active iron chelator that was approved for use in SCD in 2021. Its significant side effect is agranulocytosis, requiring weekly neutrophil counts in all patients. Gastrointestinal symptoms, arthralgia, and liver dysfunction have been reported. Deferoxamine is the first chelator introduced clinically. It is a daily subcutaneous infusion lasting for many hours or can be given intravenously for a quick iron chelation effect. It has a short half-life, which affects its efficacy. Due to deferoxamine's toxicity at low body iron levels, its use has been conservative and only started when serum ferritin levels reached 1000 ug/L [4].

Effectiveness of CDS tools in the management of SCD

CDS tools are valuable resources that aid healthcare providers in making informed decisions for patients. Examples include order sets tailored to specific conditions or patient types, recommendations based on best practices, databases that offer relevant patient information, reminders for preventive care, and alerts for potential risks or hazardous situations [35]. CDS tools help improve patient care and are very useful in SCD management. One study that evaluated the impact of CDS on pediatric transfusion practices, utilizing a user-centered design, observed a reduction in ordering errors and inappropriate transfusion rates [36]. Linton et al. also found a significant percentage increase in SCD patients receiving proper triage assignments when CDS was utilized in their study [37]. Triage nurses favored the tool, and it helped improve their triage practices for SCD patients in the emergency department. In addition, triage guideline concordance was achieved without the need for direct nursing education. Findings from another study conducted by Marinho et al. using the forced oscillation technique revealed that sickle cell anemia patients exhibited restrictive changes, reduced compliance, increased respiratory resistance, and ventilation heterogeneity. The forced oscillation technique, combined with machine learning classifiers, demonstrated good diagnostic accuracy in detecting early respiratory abnormalities, making it a valuable tool for identifying respiratory abnormalities in sickle cell anemia patients [38].

Furthermore, precision medicine was explored in prescribing codeine to pediatric patients according to their cytochrome P450 2D6 (CYP2D6) pharmacogenetics. Codeine was previously associated with postoperative deaths in children, particularly those with atypical CYP2D6 status. Integration of CDS into the electronic health records of these patients successfully limited codeine use to patients with a safe CYP2D6 metabolizer status, thereby minimizing the risk of adverse drug events and ultimately providing a model for optimizing drug therapy in specialized pediatric populations [39]. All the CDS tools cited have been shown to significantly ameliorate some major complications of SCD management, including those associated with blood transfusions and pain medications, and have also proven effective in the early detection of pulmonary complications. Therefore, adopting more CDS interventions is crucial to improving clinical outcomes and the quality of life for SCD patients.

Public health implications

SCD disproportionately affects minority United States racial populations. However, SCD patients fare slightly better in the United States than in lower-income countries due to early diagnosis through newborn screening and patients receiving prompt and appropriate treatment. In addition, there is access to specialized medical care, genetic counseling, and better living conditions. Much work remains to be done to improve the quality of life for patients in the face of a high prevalence of co-morbidities and complications like infections, acute chest syndrome, pain crisis, stroke, chronic kidney disease, pulmonary hypertension, leg ulcers, and retinopathy. Therefore, SCD patients need regular medical care and monitoring to prevent or ameliorate these complications.

PCPs are an integral and, ideally, introductory component of the healthcare team and are tasked with coordinating care with hematologists and ED providers. This is because SCD is a complex chronic disease that responds favorably to a multi-specialty treatment modality [40]. Therefore, it is prudent that PCPs get equipped with resources to deliver comprehensive and evidence-based SCD care, especially given the increasing rate at which forced migration and ongoing population dynamics are causing the disease to spread worldwide [41].

More public education is needed to raise awareness about SCD for better health outcomes and improved quality of life for patients with SCD. Our review aims to achieve this goal by examining public health interventions that have been effective for SCD management at the primary care level and novel interventions that can help improve patient outcomes. Hydroxyurea remains effective for addressing complications of vaso-occlusive crises and hemolytic anemias. There has been progress in the development of drug therapies, with recent approvals of L-glutamine, crizanlizumab, and voxelotor by the FDA. Patients with frequent vaso-occlusive complications (acute pain episodes, acute chest syndrome) may benefit from hydroxyurea, L-glutamine, and crizanlizumab. Those with increased hemolytic anemia (or possibly complications related to hemolytic anemia) may benefit from hydroxyurea and voxelotor. More significant funding should be designated for research addressing hydroxyurea's long-term benefits and potential risks, as this step would likely increase patient acceptance and desirable health outcomes.

Similarly, more studies are required before definite recommendations are made regarding the effect of voxelotor on SCD-related complications [18,19]. In addition, further research should be directed towards developing broader serotype pneumococcal vaccines, as most cases of IPD in children with SCD are due to serotypes not included in any licensed vaccines. Until a serotype-independent pneumococcal vaccine becomes available, higher-valent PCVs should consist of more serogroups to protect this highly vulnerable group of children [42]. Still, it is astute for PCPs to continue to offer currently available regimens to patients as appropriate. IPD in children with SCD remains associated with high morbidity and mortality, highlighting the importance of strict adherence to daily penicillin prophylaxis [42].

CDS tools have proven effective for improving transfusion practices, properly triaging patients, identifying respiratory abnormalities, and minimizing adverse drug reactions with pain medications among pediatric patients [36-39], emphasizing the need to incorporate more CDS approaches into diverse aspects of patient care, even at patients' homes and at the community level. Although chelation therapy helps address the accompanying complications of iron overload from chronic transfusions [34], it is noteworthy that some chelating agents have significant adverse effects. In the evolving landscape of SCD treatment, bone marrow transplant is a curative treatment option for SCD patients. Gene editing therapy is another emerging innovation to cure SCD. Clinical trials have shown promising results [43], and in the future, FDA approval could perhaps help it to eradicate SCD.

## Conclusions

This literature review examined the efficacy of various interventions in managing SCD within primary care settings, highlighting the benefits of hydroxyurea, pneumococcal vaccination, penicillin prophylaxis, iron chelation therapy, and CDC tools in enhancing patient outcomes and alleviating the burden of complications associated with this complex hematological disorder. By integrating these interventions and ensuring ongoing monitoring of their efficacy, PCPs can optimize patient care, reduce complications, and improve the overall quality of life for those living with this chronic condition. However, while the reviewed literature demonstrated positive results for these interventions, it is also important to acknowledge new promising therapies such as L-glutamine, crizanlizumab, and voxelotor, which show potential in addressing SCD complications. Additionally, gene editing therapies present innovative approaches that have the potential to improve SCD management and potentially cure the disease in the future.

## References

[1] (2023). Data & Statistics on Sickle Cell Disease. https://www.cdc.gov/ncbddd/sicklecell/data.html.

[2] Shook LM, Farrell CB, Kalinyak KA (2016). Translating sickle cell guidelines into practice for primary care providers with Project ECHO. Med Educ Online.

[3] Farid Y, Bowman NS, Lecat P (2023). Biochemistry, hemoglobin synthesis. StatPearls [Internet].

[4] Hafen BB, Sharma S (2023). Oxygen saturation. StatPearls [Internet].

[5] Solomon N, Segaran N, Badawy M (2022). Manifestations of sickle cell disorder at abdominal and pelvic imaging. Radiographics.

[6] (2023). What is Sickle Cell Disease?. Assessed 07/23/23.

[7] McKeown RE (2009). The epidemiologic transition: changing patterns of mortality and population dynamics. Am J Lifestyle Med.

[8] (2014). Evidence-Based Management of Sickle Cell Disease: Expert Panel Report, 2014 | NHLBI, NIH. Accessed July 2, 2023. Evidence-Based Management of Sickle Cell Disease: Expert Panel Report, 2014.

[9] DeBaun MR, Chou ST (2022). Transfusion in sickle cell disease: Management of complications including iron overload. UpToDate.

[10] Agrawal RK, Patel RK, Shah V, Nainiwal L, Trivedi B (2014). Hydroxyurea in sickle cell disease: drug review. Indian J Hematol Blood Transfus.

[11] Jones W, Jang A, Myers L, Dasgupta A, DeBord J (2022). Clinical pathway for vaso-occlusive pain reduces hospital admissions. J Healthc Qual.

[12] (2022). Vaccination Coverage among Adults in the United States, National Health Interview Survey, 2019-2020. https://www.cdc.gov/vaccines/imz-managers/coverage/adultvaxview/pubs-resources/vaccination-coverage-adults-2019-2020.html.

[13] (2023). Pneumococcal Vaccination: What Everyone Should Know. https://www.cdc.gov/vaccines/vpd/pneumo/public/index.html.

[14] (2023). Invasive Pneumococcal Disease among Children with and without Sickle Cell Disease in the United States, 1998-2009. https://www.cdc.gov/ncbddd/sicklecell/features/keyfinding-pneumococcal.html.

[15] Cober MP, Phelps SJ (2010). Penicillin prophylaxis in children with sickle cell disease. J Pediatr Pharmacol Ther.

[16] Rasel M, Mahboobi SK (2023). Transfusion iron overload. StatPearls [Internet].

[17] Sutton RT, Pincock D, Baumgart DC, Sadowski DC, Fedorak RN, Kroeker KI (2020). An overview of clinical decision support systems: benefits, risks, and strategies for success. NPJ Digit Med.

[18] Rai P, Ataga KI (2020). Drug therapies for the management of sickle cell disease. F1000Res.

[19] Ballas SK (2020). The evolving pharmacotherapeutic landscape for the treatment of sickle cell disease. Mediterr J Hematol Infect Dis.

[20] Rankine-Mullings AE, Nevitt SJ (2022). Hydroxyurea (hydroxycarbamide) for sickle cell disease. Cochrane Database Syst Rev.

[21] Ofakunrin AO, Oguche S, Adekola K (2020). Effectiveness and safety of hydroxyurea in the treatment of sickle cell anaemia children in Jos, north-central Nigeria. J Trop Pediatr.

[22] Neal EF, Chan J, Nguyen CD, Russell FM (2022). Factors associated with pneumococcal nasopharyngeal carriage: a systematic review. PLOS Glob Public Health.

[23] Yee ME, Lai KW, Bakshi N (2022). Bloodstream infections in children with sickle cell disease: 2010-2019. Pediatrics.

[24] (2023). Pneumococcal Disease. https://www.cdc.gov/vaccines/pubs/pinkbook/pneumo.html.

[25] Daniels CC, Rogers PD, Shelton CM (2016). A review of pneumococcal vaccines: current polysaccharide vaccine recommendations and future protein antigens. J Pediatr Pharmacol Ther.

[26] Adamkiewicz TV, Sarnaik S, Buchanan GR (2003). Invasive pneumococcal infections in children with sickle cell disease in the era of penicillin prophylaxis, antibiotic resistance, and 23-valent pneumococcal polysaccharide vaccination. J Pediatr.

[27] Adamkiewicz TV, Silk BJ, Howgate J, Baughman W, Strayhorn G, Sullivan K, Farley MM (2008). Effectiveness of the 7-valent pneumococcal conjugate vaccine in children with sickle cell disease in the first decade of life. Pediatrics.

[28] Scott NR, Mann B, Tuomanen EI, Orihuela CJ (2021). Multi-valent protein hybrid pneumococcal vaccines: a strategy for the next generation of vaccines. Vaccines (Basel).

[29] Huang L, Wasserman M, Grant L (2022). Burden of pneumococcal disease due to serotypes covered by the 13-valent and new higher-valent pneumococcal conjugate vaccines in the United States. Vaccine.

[30] Rahim MQ, Arends AM, Jacob SA (2022). Maintenance of an immunogenic response to pneumococcal vaccination in children with sickle cell disease. J Pediatr Hematol Oncol.

[31] Robinson TM, Lanzkron SM (2019). Standard definitions of pneumococcal immunity may not accurately predict protection in adults with sickle cell disease. Blood.

[32] Brown BJ, Madu A, Sangeda RZ (2021). Utilization of pneumococcal vaccine and penicillin prophylaxis in sickle cell disease in three African countries: assessment among healthcare providers in SickleInAfrica. Hemoglobin.

[33] Rankine-Mullings AE, Owusu-Ofori S (2017). Prophylactic antibiotics for preventing pneumococcal infection in children with sickle cell disease. Cochrane Database Syst Rev.

[34] Porter J, Viprakasit V, Kattamis A (2023). Iron overload and chelation. Guidelines for the Management of Transfusion Dependent Thalassaemia (TDT), 3rd edition.

[35] (2023). Clinical Decision Support. https://www.ahrq.gov/cpi/about/otherwebsites/clinical-decision-support/index.html.

[36] Orenstein EW, Rollins M, Jones J, Kandaswamy S, Boudreaux J, Carter AB, Josephson CD (2023). Influence of user-centered clinical decision support on pediatric blood product ordering errors. Blood Transfus.

[37] Linton E, Souffront K, Gordon L, Loo GT, Genes N, Glassberg J (2021). System level informatics to improve triage practices for sickle cell disease vaso-occlusive crisis: a cluster randomized controlled trial. J Emerg Nurs.

[38] Marinho CL, Maioli MC, do Amaral JL, Lopes AJ, Melo PL (2017). Respiratory resistance and reactance in adults with sickle cell anemia: correlation with functional exercise capacity and diagnostic use. PLoS One.

[39] Gammal RS, Crews KR, Haidar CE (2016). Pharmacogenetics for safe codeine use in sickle cell disease. Pediatrics.

[40] Tanabe P, Blewer AL, Bonnabeau E (2021). Dissemination of evidence-based recommendations for sickle cell disease to primary care and emergency department providers in North Carolina: a cost benefit analysis. J Health Econ Outcomes Res.

[41] Asnani MR, Quimby KR, Bennett NR, Francis DK (2016). Interventions for patients and caregivers to improve knowledge of sickle cell disease and recognition of its related complications. Cochrane Database Syst Rev.

[42] Oligbu G, Fallaha M, Pay L, Ladhani S (2019). Risk of invasive pneumococcal disease in children with sickle cell disease in the era of conjugate vaccines: a systematic review of the literature. Br J Haematol.

[43] Zarghamian P, Klermund J, Cathomen T (2022). Clinical genome editing to treat sickle cell disease-a brief update. Front Med (Lausanne).

